# A novel cell permeability assay for macromolecules

**DOI:** 10.1186/s12860-020-00321-x

**Published:** 2020-10-30

**Authors:** Yensi Flores Bueso, Sidney Walker, Jennifer Quinn, Mark Tangney

**Affiliations:** 1 CancerResearch@UCC, University College Cork, Cork, Ireland; 2 SynBioCentre, University College Cork, Cork, Ireland; 3 APC Microbiome Ireland, University College Cork, Cork, Ireland

**Keywords:** Cell permeabilisation, Biotin-streptavidin, In vitro labelling, MW marker, Host DNA depletion

## Abstract

**Background:**

Many cell permeabilisation methods to mediate internalisation of various molecules to mammalian or bacterial cells have been developed. However, no size-specific permeability assay suitable for both cell types exists.

**Results:**

We report the use of intrinsically biotinylated cell components as the target for reporter molecules for assessing permeabilisation. Due to its well-described biotin binding activity, we developed an assay using Streptavidin (SAv) as a molecular weight marker for assessing eukaryotic and prokaryotic cell internalisation, using flow cytometry as a readout. This concept was tested here as part of the development of host DNA depletion strategies for microbiome analysis of formalin-fixed (FF) samples. Host depletion (HD) strategies require differential cell permeabilisation, where mammalian cells but not bacterial cells are permeabilised, and are subsequently treated with a nuclease. Here, the internalisation of a SAv-conjugate was used as a reference for nucleases of similar dimensions. With this assay, it was possible to demonstrate that formalin fixation does not generate pores which allow the introduction of 60 KDa molecules in mammalian or bacterial membranes/envelopes. Among surfactants tested, Saponin derived from Quillaja bark showed the best selectivity for mammalian cell permeabilisation, which, when coupled with Benzonase nuclease, provided the best results for host DNA depletion, representing a new HD strategy for formalin fixed samples.

**Conclusion:**

The assay presented provides researchers with a sensitive and accessible tool for discerning membrane/cell envelop permeability for different size macromolecules.

**Supplementary Information:**

The online version contains supplementary material available at 10.1186/s12860-020-00321-x.

## Background

The role that different macromolecules play within the cellular milieu is routinely studied with in vitro techniques that involve their ex vitro modification (labelling) and subsequent cellular internalisation [1]. Since the membrane of live mammalian cells is virtually impermeable against polar and charged molecules with a molecular weight (MW) larger than ~ 118 Da [2, 3], and the outer membrane of Gram-negative (G-) bacteria is only permeable to hydrophilic molecules smaller than ~ 600 Da [4], the internalisation of biomolecules must be artificially induced. For in vitro studies, this is achieved by permeabilising the cell membrane/envelope [5]. A plethora of permeabilisation methods have been developed for mammalian cells [6], and to a lesser extent for bacteria [7], including solvents (Alcohol, acetone), detergents (Triton X-100, Saponins), toxins (crotalicidin, streptolysin-O), enzymes (Lysozyme, Proteinases) and even hydrochloric acid [5, 8, 9]. However, no single method has yet been found that is suitable for multiple cell types or biomolecules. Additionally, the evaluation of their efficacy is established empirically, on a case-by-case basis, which is laborious and often irreproducible. This can be partially attributed to the restricted methods available, which can only assess membrane permeability for small molecule membrane impermeable dyes, such as SYTOX®, YO-PRO®, Propidium Iodide (PI) (< 1 KDa) and 7-amino-actinomycin D (7-AAD) (1.3 KDa) [10, 11]. Methods for examining cell permeability to large molecules are not standardised. Thus, we hypothesised that an accessible method enabling the assessment of cell permeabilisation, in terms of MW cut-offs and applicable to different cell types, could facilitate cell permeabilisation assessment.

Such a method could be enabled via the exploitation of intrinsic cellular motifs as targets for reporter molecules of a given size. Analogous assays for examining cell permeability to small molecules utilise cellular DNA as the target for reporters (DNA-binding small molecule dyes such as PI and 7-AAD. For examination of macromolecules, we have exploited biotin as the target, an intrinsic and essential co-factor for many enzymes in all domains of life, from prokaryotes [12] to eukaryotes [13]. SAv binds to biotin in one of the strongest (K_d_ ~ 10^− 15^ M), highly specific, and rapid interactions observed in nature [14], and has been exploited for many purposes [15]. SAv is a globular tetramer, with a MW of ~ 52 KDa, and a dimension of 5 nm, with each monomer able to interact with a biotin molecule [16]. With this information, we developed a new assay to assess permeabilisation, using flow cytometry as a readout. Here, the detection of naturally biotinylated intracellular proteins by SAv serves as a MW marker for cell internalisation. This assay can be easily adapted for research on different biomolecules in eukaryotic or prokaryotic cells and is scalable to high-throughput settings.

The in vitro study of cells often requires their fixation [17]. Formaldehyde is the most widely adopted fixative, since it preserves the overall cellular structure, although partially permeabilising cellular membranes/envelopes [18, 19]. Formalin Fixed, Paraffin Embedded (FFPE) archives could provide access to an unprecedented number of samples for the study of the human microbiome - the microbial communities living within the human body [20]. A key consideration however, similar to non-fixed (NF) samples, is that these samples have a low microbial biomass [21]. In low biomass samples, microbial sequencing analysis is impaired by the high background of human (host) DNA masking bacterial DNA. To address this issue, several host depletion strategies been published for non-fixed samples, with different level of success. Most of these strategies involve the permeabilisation of host cells but not bacterial cells, with a subsequent treatment with a DNase nuclease to digest exposed host DNA [22]. However, the suitability of these strategies has yet to be investigated for FF samples and to date, no host depletion strategies have been specifically developed for this sample type.

With this study we aimed to inform future development of host depletion strategies for FF samples by assessing: *(1)* the permeabilisation state of FF bacterial and mammalian cells, and *(2)* the permeabilisation efficacy of non-ionic surfactants for FF cells as potential permeabilisation agents. A SAv-conjugate with similar dimensions to DNase was used as a marker for cell internalisation, allowing a clear assessment of the surfactants’ permeabilisation efficacy for nucleases. The assay was validated by assessing nuclease activity with qPCR and flow cytometry.

## Results

### Study overview

The aim of this study was to develop an accessible assay for assessing cell permeabilisation for in vitro analysis, such that it is applicable to eukaryotic or prokaryotic cells, and for multiple macromolecules. To achieve this, we hypothesised that: *1)* permeabilisation could be defined in terms of a general macromolecule size feature, such as MW. *2)* An intrinsic cellular factor could serve as an internalisation marker for molecules of different MW.

To test these hypotheses, we sought an easily accessible internalisation marker and found SAv to be an ideal candidate. As such, we used SAv to design an easily accessible assay that, following permeabilisation, only requires labelling of cells with the SAv-conjugate of choice. Labelled cells can be analysed by flow cytometry (as performed here) or by another suitable SAv detection method available to researchers. The workflow for this assay is illustrated in sFigure 1a. In this setting, the SAv-conjugate will only bind to cell intrinsic biotin if the membrane/envelope permeabilisation has been successful. Thus, the internalisation of molecules with similar MWs should be feasible with the same permeabilisation method. This approach was tested here in bacterial (*E. coli*) and mammalian (4 T1) cells, with 4 different permeabilisation agents and 2 different SAv conjugates. In addition, the assay was validated with a functional experiment assessing the internalisation and activity of a nuclease with dimensions similar to those of the SAv conjugate tested, as described below.

### SAv allows the assessment of membrane permeabilisation

**Fig. 1:**
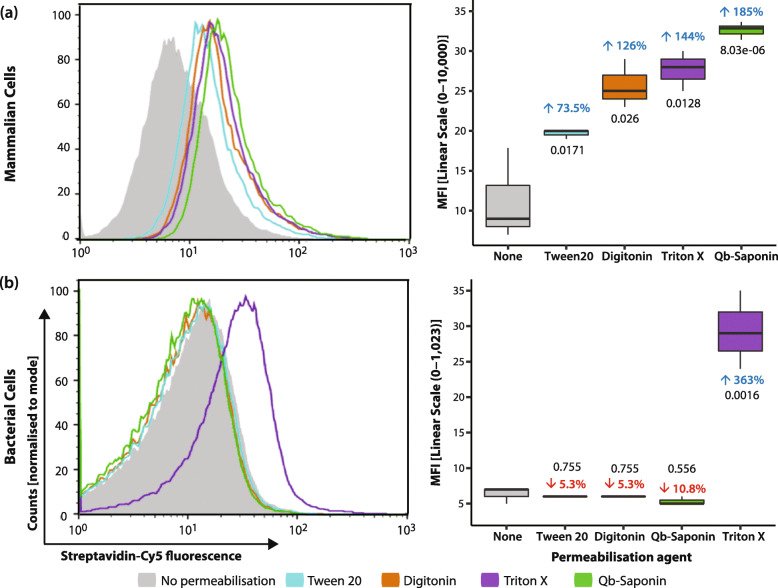
Membrane permeabilisation. Cell permeabilisation is measured by the internalisation of SAv-Cy5 for (**a**) 4 T1 cells and (**b**) *E. coli*. (Left) Histograms showing Cy5+ maximum fluorescence intensity (*n* = $\overline{\mathrm{x}}$ 6). (Right) Box plot showing median fluorescence intensity. Deviation (%) from impermeabilised shown above each box in blue/red and *p*-values are shown in black. In all cases *n* = 6

Streptavidin-Cy5 served as the cell internalisation marker for Benzonase, a dimeric nuclease, with a MW of 60 KDa [23]. Each monomer corresponds to the dimensions of DNase I – a compact monomer with a MW of ~ 30 KDa and dimensions of 4.6 × 4 × 3.5 nm [24]. Benzonase was the most cost effective enzyme of 6 DNases examined (sFigure 2b), it exhibited the highest levels of activity per quantity of enzyme used. The permeabilisation capability of FF and 4 non-ionic detergents for 60 KDa molecules was assessed in 4 T1 and *E. coli* cells following the workflow in sFigure 1a. As shown in Fig. 1a, impermeabilised FF 4 T1 cells showed significantly less SAv-Cy5 fluorescence than those exposed to detergents, while only *E. coli* cells (Fig. 1b) treated with Triton-X were permeabilised, as evidenced by a 363X increase in fluorescence (*p* < 0.001). This indicates that fixation does not permeabilise cells to large molecules. Among the detergents tested, Quillaja bark Saponin (Qb-Saponin) (displayed the highest membrane selectivity for Sav in 4 T1 cells, with a 186X (*p* < 0.001) increase in fluorescence, however no significant fluorescence change for *E. coli* was detected (*p* > 0.05). This held true for *E. coli* cells exposed to higher Qb-Saponin concentrations (sFigure 2a). When a larger molecule was examined in 4 T1 cells - 360 KDa Streptavidin Phycoerythrin (SAv-PE) – internalisation was much lower (2–25%) than that observed for SAv-Cy5, as expected, although patterns of detergent efficacy varied, with internalisation only detectable for Digitonin (10.8%) and Qb-Saponin (25%) (sFigure 3).

### Validation of the permeabilisation strategy by nuclease activity

**Fig. 2:**
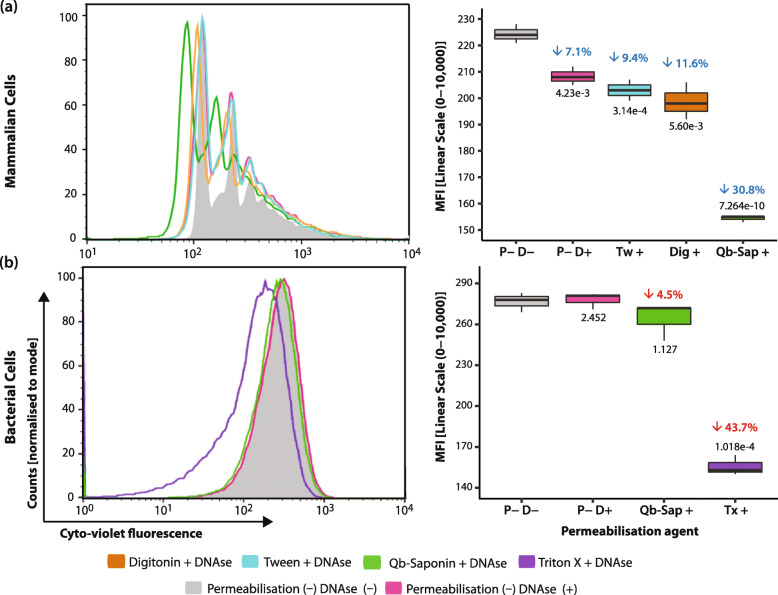
DNA depletion. DNA depletion is measured here by a reduction in fluorescence of the double-stranded DNA intercalating dye CytoPhase, measured for (**a**) 4 T1 cells and (**b**) *E. coli*. (Left) Histograms showing the maximum fluorescence intensity for CytoPhase+ cells. (Right) Box plot showing median fluorescence intensity. Deviation (%) from impermeabilised DNase- shown above each box in blue/red and p-values are shown in black. In all cases *n* = 6

**Fig. 3:**
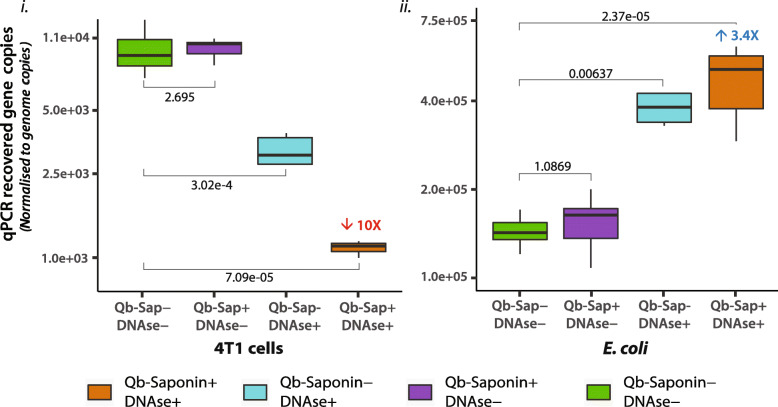
Quantifying DNA Depletion. DNA depletion measured by a reduction in the qPCR recovery of genomes of (**i**) 1 × 10^5^ 4 T1 cells and (**ii**) 1 × 10^6^ *E. coli* from a mixed cell suspension treated or not with Qb-Saponin and Benzonase. Deviation (%) from impermeabilised DNase- shown above each box in blue/red and p-values are shown in black. In all cases *n* = 6

Nuclease activity in permeabilised cells was tested by measuring the fluorescence emitted by a cell permeable, double-stranded DNA intercalating dye (CytoPhase Violet), after treatment with a permeabilisation (P+) agent and Benzonase (sFigure 1b). Following membrane permeabilisation, the nuclease can passively diffuse through the cytoplasm and pores in the nuclear membrane [25]. A reduction in CytoPhase signal is indicative of a reduction in DNA content, and thus higher nuclease activity. Results presented in Fig. 2 reflect those in Fig. 1, with the most significant CytoPhase signal reduction (30.8%, *p* < 0.001) observed in 4 T1 cells permeabilised with Qb-Saponin. Conversely, Qb-saponin treatment did not lead to any significant decrease in fluorescence for *E. coli* (4.5% decrease, *p* > 0.05), while treatment with Triton-X (P+ DNAse+ control), showed the greatest decrease 43.7% (*p* < 0.001). It was also noticeable that harvesting or pre-treatments did not significantly affect the integrity of the *E. coli* cells envelope, as impermeabilised cells exposed to Benzonase did not show a significant decrease in CytoPhase signal. These results were verified by qPCR, whereby a mixed FF cell population, containing 1 × 10^7^ *E. coli* and 1 × 10^6^ 4 T1 cells*,* were exposed to the Host DNA depletion (HD) strategy, after which cells were harvested and DNA purified. Eluted DNA was analysed by qPCR. As seen in Fig. 3(i), for 4 T1 cells, the quantity of genes (normalised to genome copies) retrieved after HD were reduced by 10-fold (*p* < 0.01), which suggests that approximately 90% of the cells were permeabilised and their DNA content digested. Due to the reduced interference of mammalian DNA, HD treatment allowed for a higher (truer) representation of bacterial DNA, which exhibited a 3X (*p* < 0.01) increase in the number of genomes recovered (Fig. 3(ii)). Altogether, these results validate the permeabilisation assessment strategy and confirm that Qb-Saponin shows the best cell selective permeabilisation capacity.

## Discussion

In this study, we developed an assay to evaluate cell permeabilisation of biomolecules, which may reduce the case-by-case evaluation and optimisation of permeabilisation strategies. Since cell internalisation is directly proportional to molecule size [2], our results suggest that evaluation of permeabilisation can be achieved by establishing MW cut-offs, where MW internalisation markers indicate the permeabilisation efficiency expected for biomolecules of similar size. For this purpose, a MW marker should fulfil two key criteria: 1) it is applicable to all cell types, and 2) Size modulation, that the size of the marker can be adjusted to different sizes investigated. Fitting these criteria is the Biotin-SAv interaction, since Biotin is an intrinsic cell co-factor present across all domains of life [12, 13], and thus allows the evaluation of permeabilisation treatments in a wide variety of cells, as disparate as the Gram-negative *E. coli* and 4 T1 murine cancer cells examined here. This is exemplified in Fig. 1, where Qb-saponin and Tween-20 proved effective at permeabilising 4 T1 cells but not *E. coli*. In addition, different forms of SAv (monomeric, dimeric) [16], coupled with the plethora of available SAv conjugates of different MW, allow for size modulation, as demonstrated in Fig. 1 for SAv-Cy5 (60KDa) and sFigure 3 for SAv-PE (360 KDa).

By implementing a MW internalisation marker, the proposed approach defines the efficiency at which biomolecules of different sizes can be internalised into the cells tested. As shown in Figs. 1 and s2, the permeabilisation efficiency of Qb-Saponin treatment in 4 T1 cells was < 25% for the 360 KDa size molecule, and > 70% for the 60 KDa molecule. This data suggests that permeabilisation efficiency can be inferred based on the reporter molecule size. Reporter molecule size may be an important factor which is overlooked and may contribute to the controversial efficiencies reported for available permeabilisation protocols. As a result, researchers must optimise protocols for each cell type and biomolecule studied, as reflected by the plethora of biomolecule and cell type specific permeabilisation methods published to date [7, 26, 27].

To reduce the need for this case-by-case approach, the assay developed in this study provides an accessible platform applicable for different cell types and biomolecules, tested in bacteria and mammalian cells. Here, the MW-cut off for a permeabilisation treatment in a cell type is established by using markers with the MW of interest. All biomolecules with a MW equal to or below that of the marker subjected to the specified treatment will exhibit the same degree of permeability as the marker, without the need for optimisation. This was demonstrated and validated here, as SAv-Cy5 could correctly predict whether a nuclease of comparable dimensions could gain entry to the targeted cells (Figs. 1 & 2).

This protocol was validated in an evaluation of potential permeabilisation agents for host depletion strategies in FF samples for microbiome analysis. Structural differences between mammalian membranes and G- bacterial envelopes inform the choice of permeabilisation agent [28]. While both the mammalian and the G- bacterial outer membrane (OM) are mostly composed of phospholipids [29, 30], the mammalian bilayer also contains variable contents of cholesterol [29, 31] and the OM of G- bacteria contains a tightly packed lipopolysaccharide (LPS) structure that protects it against surfactants [32, 33]. This favours the use of non-ionic detergents [28], which exclusively target cholesterol (Saponins) [34, 35]. Different levels of success have been reported for host depletion strategies in non-fixed (NF) samples [36], but not for FF samples. While the underlying principles for guiding the choice of permeabilisation agent are valid for NF samples, they do not always apply to FF samples [19, 37]. This was investigated here, and it is clear from the results of this study that formalin fixation does not induce pores allowing the entrance of 60 KDa molecules in both mammalian and G- bacterial cells. This highlights the need for bacterial lysis strategies in the processing FF samples for microbial analyses.

It was also proven here that whilst all permeabilisation agents tested induced pores in mammalian cells, their efficiencies varied, with Qb-saponin displaying the highest mammalian cell selective permeabilisation capability. This was confirmed by HD experiments, where a marked reduction in DNA quantity was observed after treatment with Qb-saponin + Benzonase for host (4 T1) cells only (Figs. 2 & 3). These results are supported by recently-published evidence of Qb-saponin [23, 27] and Benzonase [22, 36] treatment on non-fixed samples [22, 38]. This information can provide foundational knowledge for the development of host depletion strategies for FFPE tissues and assist in unlocking the potential of FFPE samples, providing researchers with unprecedented access to samples. Furthermore, the permeabilisation assessment protocol presented here can be used to test different bacterial strains against different permeabilisation agents. An essential requirement for the development of host depletion strategies for this sample type.

## Conclusions

Here, we have presented an accessible assay for assessing cell permeabilisation, applicable for multiple biomolecules in bacteria and mammalian cells. By defining permeabilisation in terms of MW-cut offs and identifying a MW marker that is intrinsic to all types of cells, this assay has the potential to be used in a broad range of cell types. Given the nature of the analyses and the uniformity of the marker used across cell types, this assay is well suited for scaling to high-throughput experiments, allowing in-parallel permeability assessment. Furthermore, while not explored in this study, the protocol presented could be adapted for the study of live cells or cells fixed with other fixation strategies.

## Methods

### Cell culture

*Mus musculus* mammary gland cancer cells (4 T1) were grown at 37 °C 5% CO2, in Roswell Park Memorial Institute (RPMI) media supplemented with 10% v/v Foetal Bovine Serum, 100 U/mL penicillin and 100 μg/mL of streptomycin (ThermoFisher). The cells were harvested with 0.5 ml/10cm^2^ trypsin by centrifugation at 180 x g, washed with Phosphate Buffer Saline (PBS), and counted with a NucleoCounter® NC-100™ (chemometect, Copenhagen) following manufacturer’s instructions. The cells were fixed in 40 ml of 4% w/v buffered formalin for 24 h at room temperature (RT).

### Bacterial growth conditions

*Escherichia. coli* K12 MG1655 carrying a P16Lux plasmid [39] was grown aerobically at 37 °C to an OD_600_ of 0.8 in Luria-Bertani (LB) medium supplemented with 300 μg/ml Erythromycin and harvested by centrifugation at 3000 x g, for 10 min at 4 °C, suspended to a 2X concentration in 4% w/v buffered formalin for 24 h at RT.

### Counting fixed bacterial cells

Bacterial cell suspensions were counted following the instructions of the bacterial counting kit (Invitrogen). In brief, after fixation, a 10% aliquot was taken from this suspension and serially diluted (100X) with filtered sterilised 0.15 M NaCl solution to obtain a cell density of approximately 1 × 10^6^ cells in 989 μl of NaCl. Bacterial cells were stained with 1 μl of Syto® BC and 10 μl (1X 10^6^) of counting beads were added to the suspension. Cells were counted in an LSR II Flow Cytometer (BD Biosciences, NJ, USA). The acquisition trigger was set to side scatter and set to 800.

### Membrane permeabilisation assay

After fixation, 8 × 10^7^ 4 T1 cells were harvested at 180 x g for 10 min, washed once with 20 ml of Tris Buffer Saline (TBS) (50 mM Tris, 150 mM NaCl, pH 7.6), and suspended to a final density of 2.5 × 10^6^ cells per ml in TBS. Similarly, 5 × 10^9^ *E. coli* cells, were harvested at 300 x g for 10 min, washed once with 20 ml of TBS and suspended to a final density of 2.5 × 10^7^ cell per ml in TBS. 500 μl of the cell suspensions were aliquoted into 1.5 ml tubes and treated with a permeabilisation agent. Permeabilisation agents tested were: Triton X-100 (0.1% v/v), Tween-20 (0.2% v/v), Saponin (from Quillaia bark) (0.1% w/v) (S4521), Digitonin (D141) (0.5% w/v). All were acquired from Sigma-Aldrich. Concentrations used were derived from several protocols [6].

The cells were permeabilised for 25 min, at 25 °C, shaking at 500 rpm. Permeabilised cells were washed once with TBS (centrifugation speeds as above) and blocked on ice with TBS + 1% w/v Bovine Serum Albumin (BSA) for 30 min. Blocked cells were exposed to 0.75 μg of Cyanine-5 (Cy5) or Phycoerythrin (PE) labelled Streptavidin (SAv-Cy5, MW = 60 KDa or SAv-PE, MW = 360 KDa) (Biolegend, CA, USA) for 30 min at 25 °C, shaking at 280 rpm. Cells were washed with 1 ml of 0.15 M NaCl solution and resuspended in 350 μl of the same solution for analysis. Bacterial cells were also labelled with 1 μl of Syto® BC (Invitrogen) for 5 min and analysed by flow cytometry in a BD LSRII. 4 T1 cells were identified and gated based on their Forward/Side scatter and *E. coli* cells were detected using the 488–1 (Fluorescein isothiocyanate - FITC), 525/50 filter for Syto® BC and gated using the side scatter. Cy5 positive cells were detected with the red 670/14 filter. PE positive cells were detected with the yellow/green 780/60 filter. For each experimental replicate, 3 × 10,000 events were recorded for 4 T1 cells and 3 × 100,000 for bacteria.

### DNase screening

A screen to identify the DNase with the highest DNA depleting activity in a reaction buffer containing Qb-Saponin was performed. DNases tested were as follows: Recombinant DNase I [1-2 U, 1 μl] (Sigma-Aldrich), Turbo DNase [2 U, 1 μl] (Thermo-Fisher), Molysis DNase [2 μl] (Molzyme GmbH & Co, Bremen, Germany), RQ1 DNase [20 U, 20 μl] (Promega), Benzonase [75 U, 0.3 μl] (Sigma-Aldrich). 5 × 10^6^ 4 T1 cells, FF for 48 h were treated with 0.2% w/v Qb-Saponin and the DNases tested. Reactions were set in reaction buffers provided or suggested by the supplier for 20 min at 37 °C. The reaction was stopped by either: the addition of Ethylenediaminetetraacetic acid (EDTA) for Benzonase, the supplied reaction Stop Buffer, or by incubating at 75 °C (DNAse I). Cells were then subject to DNA purification with the QIAamp DNA Mini Kit (QIAGEN). DNA yield was measured with Qubit™ dsDNA HS Assay Kit (Invitrogen). All reactions were performed in triplicate. A no-DNAse control was included. This was incubated under the same conditions with buffer supplied for DNAse I, but without the nuclease.

### Quillaja bark Saponin titration

Different w/v concentrations (0.1, 0.25, 0.5, 1%) were tested in 1 × 10^6^ *E. coli cells* that were fixed, washed, permeabilised, blocked and imaged as described for membrane permeabilisation assay.

### DNA depletion assay

Cells were fixed, washed and permeabilised as described for the membrane permeabilisation assay. 2.5 × 10^5^ 4 T1 or 2.5 × 10^6^ *E. coli* cells were permeabilised, blocked with 500 μl of 1% w/v BSA in TBS+ MgCl_2_ (20 mM Tris-HCL, 20 mM NaCl, 2 mM MgCl2, pH 8) for 30 min on ice. Blocked cells were treated with 1.5 μl (≥ 375 units) of Benzonase nuclease (Sigma-Aldrich) for 30 min at 37 °C, shaking at 360 rpm. Treatment was stopped by the addition of 100 mM EDTA. The cells were washed once with TBS and suspended in 0.15 M NaCl, where they were stained with 10 μM CytoPhase Violet (Biolegend) for 1 h at RT, shaking at 200 rpm in the dark. Bacterial cells were labelled with 100 μM of BacLight red (Invitrogen) for 15 min at RT, shaking at 200 rpm and analysed by flow cytometry. 4 T1 cells were identified and gated based on their Forward/Side scatter and *E. coli* cells were detected using the 561 laser (Yellow/Green) 660/20 filter for BacLight red and gated using the side scatter. CytoPhase+ cells were detected with the 355 (UV) laser and 450/50 filter.

### Confirmation of host depletion (HD) strategy

The efficacy of the combined treatment was verified by qPCR in DNA purified from a mixed cell suspension, consisting of 1 × 10^7^ *E. coli* cells and 1 × 10^4^ 4 T1 cells. Cells were incubated for 30 min at 37 °C, shaking at 360 rpm in TBS or the optimised HD buffer (0.2%w/v Qb-saponin, in TBS + MgCl_2_ (20 mM Tris-HCL, 20 mM NaCl, 2 mM MgCl_2_), pH 8) with or without 500 U of Benzonase. The treated cells were then processed for DNA purification following instructions of the QIAamp DNA FFPE Tissue Kit (QIAGEN) and the purified DNA analysed by qPCR.

### Quantitative PCR (qPCR)

Reactions were prepared using LUNA Universal qPCR master mix (NEB, USA) and 0.25 μM of each primer (Table 1). The thermal profile included an initial denaturation of 1 min at 95 °C, followed by 40 cycles of denaturation at 95 °C × 10 s, annealing for 15 s at the temperature specified by NEB’s annealing temperature (Ta) calculator for Hot Start Taq, followed by 20–40 s of extension at 68 °C. For each assay, a 5-point standard curve was made from log_10_ dilutions of gene blocks corresponding to species-specific genetic regions (Table 1), using an initial concentration of 10^7^ copies. Primers and gene-blocks were acquired from IDT (Coralville, USA). Efficiency between 95 and 105% and R-square values > 0.995 were deemed as acceptable. All samples were run in triplicate.

**Table 1:** Primers used for qPCR analysis

Strain/Cell line	Gene/Accession No	Primer sequence	F/R	Product size (bp)
*E coli* MG1655 [CP032667]	IS5-like element IS5 family transposase AYG17556.1 [CP032667: 230175–231,191]	5’GCC GAA CTG TCG CTT GAT GA	F	217
		5’ATT TGT CTC AGC CGA TGC CG	R	
4 T1 cells [ATCC® CRL-539™] *Mus musculus* [10090]	BetaActin AC144818.4 [NC000071.6: 73696–73,082]	5’GAT TAC TGC TCT GGC TCC TAG	F	147
		5’GAC TCA TCG TAC TCC TGC TTG	R	

### Statistical analyses

Flow cytometry data was exported and analysed in FlowJo (BD, UK) and raw data exported to R. All statistical testing and visualisation were performed in the R environment (v3.6.3). Tests of normality were performed using the Shapiro-Wilk test. Tests of means were performed using paired samples T-Tests and Wilcoxon Signed Rank Tests as appropriate. The false discovery rate was controlled using the Bonferroni procedure. Data visualisation was performed using GGplot2 package (v3.2.1).

## Supplementary Information

**Additional file 1: Figure S1.** Outline of the experimental workflow. (a) Permeabilisation assay. Cells were fixed in formalin for 24 h, after which they were exposed to a permeabilisation agent for 25 min. Reactive formalin groups were blocked with BSA for 30 min. After this the cells were labelled with the SAv conjugate for 30 min. Excess SAv conjugate was washed away and cells analysed by flow cytometry. (b) Nuclease assay. Fixed cells were permeabilised for 25 min, after which they were treated with Benzonase nuclease for 30 min. Cells were labelled with CytoPhase violet for 1 h and analysed by flow cytometry.

**Additional file 2: Figure S2.** Optimising host DNA depletion. (a) Saponin titration. Histogram for Cy5 fluorescence intensity. *E. coli* cells were permeabilised with increasing concentrations of Saponin. Saponin treated cells showed no increase in fluorescence intensity even with 10X higher concentrations. (b) DNAse screen. Five commercially available DNAses were tested for their capacity to deplete DNA from 5 × 10^6^ FF 4 T1 cells. Bar plot shows DNA yield after DNA purification. Benzonase was the most cost-effective strategy.

**Additional file 3: Figure S3.** Internalisation of a 360KDa molecule. 4 T1 cells were permeabilised and labelled with SAv-PE (360 KDa). An overnight incubation with Triton X served as positive control (top right). With all permeabilisation agents tested for SAv-Cy5 (60 KDa) the overall increase in signal for SAv-PE was much lower than that observed for SAv-Cy5. Under the studied conditions (30 min permeabilisation) larger pores are induced by Saponin (25%) and Digitonin (10.8%), while the signal from cells treated with Tween 20 (6%) and Triton X (2%) resembled that of untreated cells (3.4% SAv-PE+).

## Data Availability

All data generated or analysed during this study are included in this published article. However, raw flow cytometry data is available from the corresponding author on reasonable request.

## Abbreviations

BSA: Bovine Serum Albumin
Cy5: Cyanine 5
DNAse+: DNAse positive
*E. coli*: *Escherichia coli*
EDTA: Ethylenediaminetetraacetic acid
FBS: Foetal Bovine Serum
FF: Formalin fixed
FFPE: Formalin Fixed Paraffin Embedded
FITC: Fluorescein isothiocyanate
G-: Gram negative
HD: Host DNA Depletion
KDa: Kilodalton
LB: Luria-Bertani
LPS: Lipopolysaccharide
MFI: Fluorescence Intensity normalised to mode
MgCl_2_: Magnesium chloride
MW: Molecular weight
NaCl: Sodium chloride
NF: Non-fixed
OD_600_: Optical density at 600 nm
P +: Permeabilisation positive
PBS: Phosphate-buffered saline
PE: Phycoerythrin
PI: Propidium Iodide
Qb-Saponin: Quillaja bark Saponin
qPCR: Quantitative Polymerase Chain Reaction
RPMI: Roswell Park Memorial Institute
RT: Room temperature (25 °C)
SAv: Streptavidin
Ta: Annealing temperature
TBS: Tris-buffered saline
Tris-HCl: Trisaminomethane hydrochloride
7-AAD: 7-amino-actinomycin D
