# Supplementary material for: A novel cell permeability assay for macromolecules
Source: BMC Mol Cell Biol. 2020 Oct 30;21:75. doi: 10.1186/s12860-020-00321-x (PMC7602297; doi:10.1186/s12860-020-00321-x)
Supplement: Supplementary file 1 — Additional file 1: Figure S1. Outline of the experimental workflow. (a) Permeabilisation assay. Cells were fixed in formalin for 24 h, after which they were exposed to a permeabilisation agent for 25 min. Reactive formalin groups were blocked with BSA for 30 min. After this the cells were labelled with the SAv conjugate for 30 min. Excess SAv conjugate was washed away and cells analysed by flow cytometry. (b) Nuclease assay. Fixed cells were permeabilised for 25 min, after which they were treated with Benzonase nuclease for 30 min. Cells were labelled with CytoPhase violet for 1 h and analysed by flow cytometry. [file 12860_2020_321_MOESM1_ESM.pptx]

## Slide 1
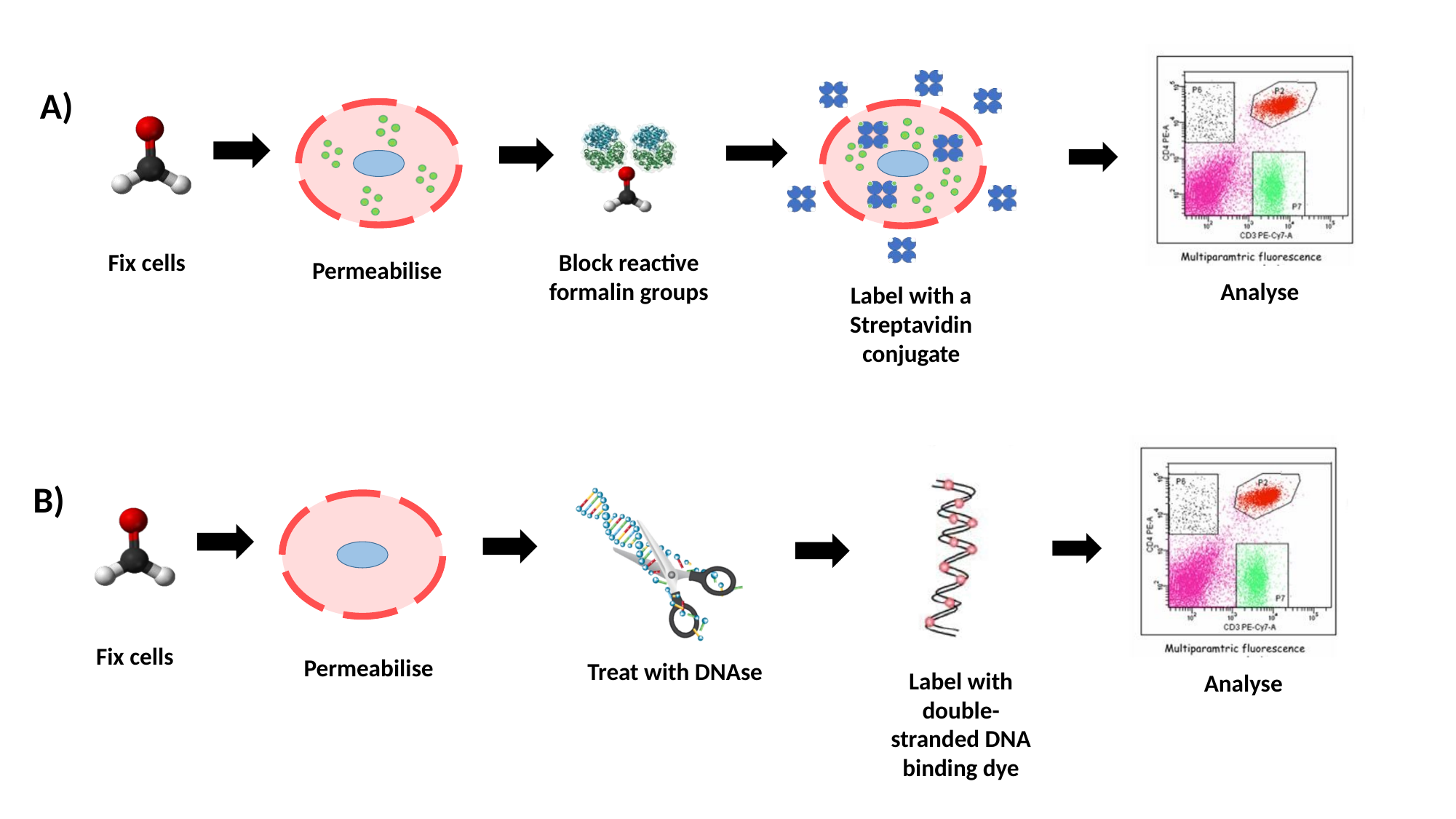

A)
Fix cells
Block reactive formalin groups
Permeabilise
Analyse
Label with a Streptavidin conjugate
B)
Fix cells
Permeabilise
Treat with DNAse
Label with double-stranded DNA binding dye
Analyse
