# Supplementary figures and images for: A novel cell permeability assay for macromolecules
Source: BMC Mol Cell Biol. 2020 Oct 30;21:75. doi: 10.1186/s12860-020-00321-x (PMC7602297; doi:10.1186/s12860-020-00321-x)

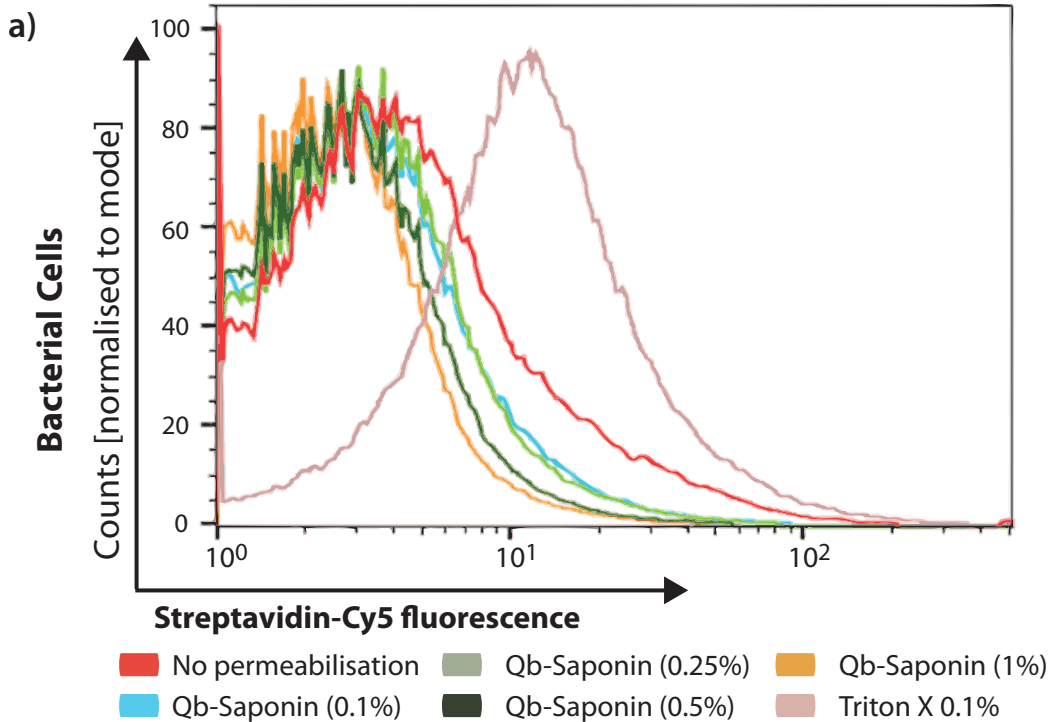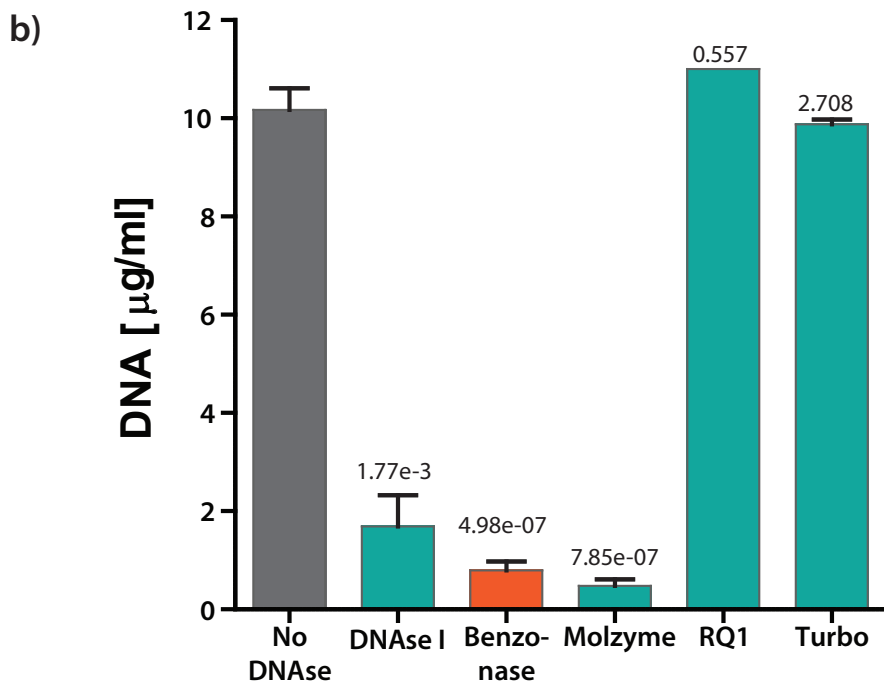

Supplement: Supplementary file 2 — Additional file 2: Figure S2. Optimising host DNA depletion. (a) Saponin titration. Histogram for Cy5 fluorescence intensity. E. coli cells were permeabilised with increasing concentrations of Saponin. Saponin treated cells showed no increase in fluorescence intensity even with 10X higher concentrations. (b) DNAse screen. Five commercially available DNAses were tested for their capacity to deplete DNA from 5 × 10^6^ FF 4 T1 cells. Bar plot shows DNA yield after DNA purification. Benzonase was the most cost-effective strategy. [file 12860_2020_321_MOESM2_ESM.pdf]
