# Supplementary material for: A novel cell permeability assay for macromolecules
Source: BMC Mol Cell Biol. 2020 Oct 30;21:75. doi: 10.1186/s12860-020-00321-x (PMC7602297; doi:10.1186/s12860-020-00321-x)
Supplement: Supplementary file 3 — Additional file 3: Figure S3. Internalisation of a 360KDa molecule. 4 T1 cells were permeabilised and labelled with SAv-PE (360 KDa). An overnight incubation with Triton X served as positive control (top right). With all permeabilisation agents tested for SAv-Cy5 (60 KDa) the overall increase in signal for SAv-PE was much lower than that observed for SAv-Cy5. Under the studied conditions (30 min permeabilisation) larger pores are induced by Saponin (25%) and Digitonin (10.8%), while the signal from cells treated with Tween 20 (6%) and Triton X (2%) resembled that of untreated cells (3.4% SAv-PE+). [file 12860_2020_321_MOESM3_ESM.pptx]

## Slide 1
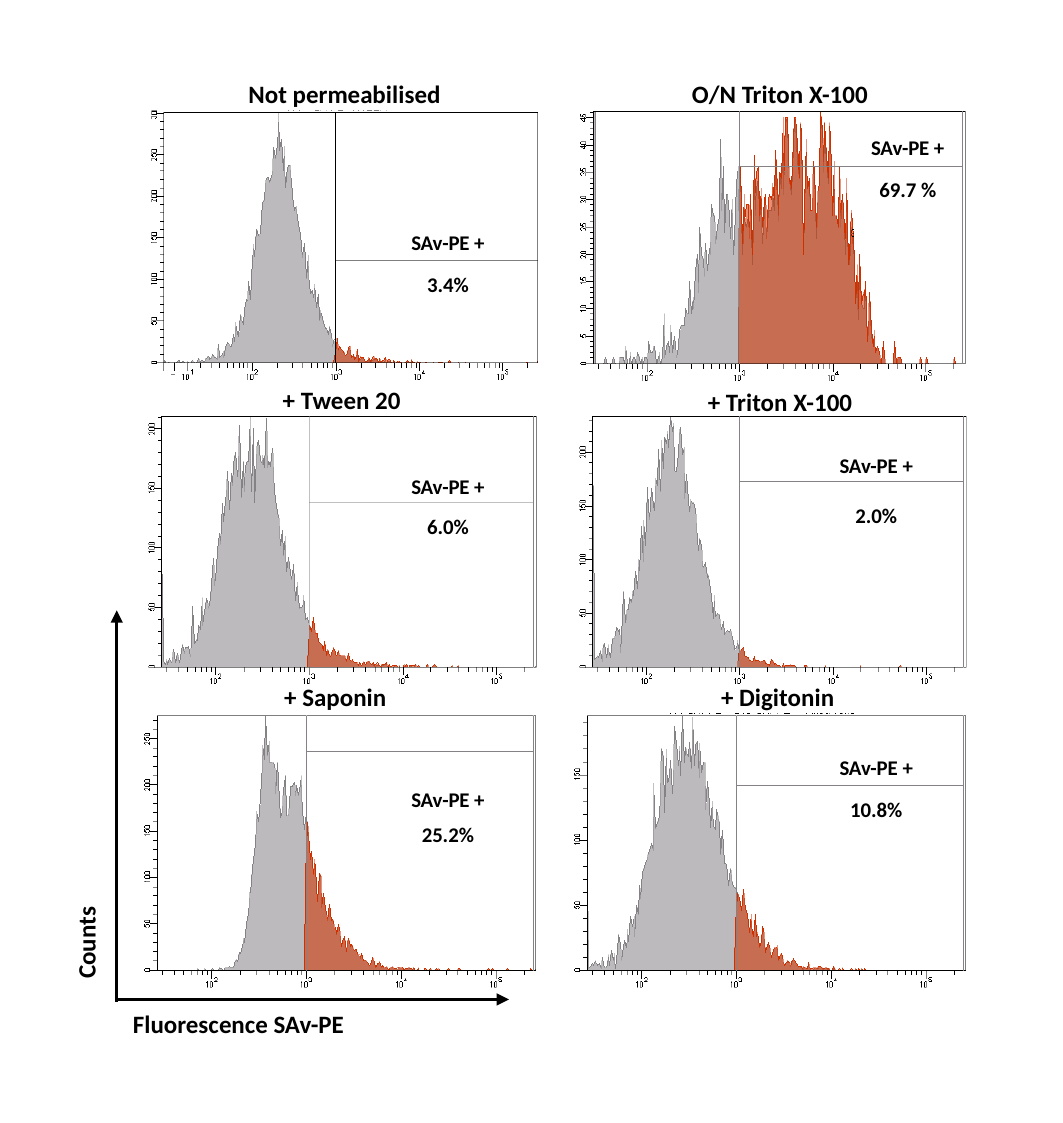

Not permeabilised
 O/N Triton X-100
SAv-PE +
69.7 %
SAv-PE +
3.4%
 + Tween 20
 + Triton X-100
SAv-PE +
SAv-PE +
2.0%
6.0%
 + Digitonin
 + Saponin
SAv-PE +
SAv-PE +
10.8%
25.2%
SAv-PE +
Counts
Fluorescence SAv-PE
